# Anti-proliferative therapy for HIV cure: a compound interest approach

**DOI:** 10.1038/s41598-017-04160-3

**Published:** 2017-06-21

**Authors:** Daniel B. Reeves, Elizabeth R. Duke, Sean M. Hughes, Martin Prlic, Florian Hladik, Joshua T. Schiffer

**Affiliations:** 1 Fred Hutchinson Cancer Research Center, Vaccine and Infectious Diseases Division, Seattle, WA 98109 USA; 2 University of Washington, Department of Medicine, Seattle, WA 98195 USA; 3 University of Washington, Departments of Obstetrics and Gynecology, Seattle, WA 98195 USA; 4 University of Washington, Department of Global Health, Seattle, WA 98105 USA; 5 Fred Hutchinson Cancer Research Center, Clinical Research Division, Seattle, WA 98109 USA

## Abstract

In the era of antiretroviral therapy (ART), HIV-1 infection is no longer tantamount to early death. Yet the benefits of treatment are available only to those who can access, afford, and tolerate taking daily pills. True cure is challenged by HIV latency, the ability of chromosomally integrated virus to persist within memory CD4^+^ T cells in a non-replicative state and activate when ART is discontinued. Using a mathematical model of HIV dynamics, we demonstrate that treatment strategies offering modest but continual enhancement of reservoir clearance rates result in faster cure than abrupt, one-time reductions in reservoir size. We frame this concept in terms of compounding interest: small changes in interest rate drastically improve returns over time. On ART, latent cell proliferation rates are orders of magnitude larger than activation and new infection rates. Contingent on subtypes of cells that may make up the reservoir and their respective proliferation rates, our model predicts that coupling clinically available, anti-proliferative therapies with ART could result in functional cure within 2–10 years rather than several decades on ART alone.

## Introduction

The most significant accomplishment in HIV medicine is the suppression of viral replication and prevention of AIDS with antiretroviral therapy (ART). However, HIV cure remains elusive due to viral latency, the ability of integrated virus to persist for decades within CD4^+^ T cells in a latent state. When ART is discontinued, latent cells soon activate, and virus rebounds^1, 2^. HIV cure strategies aim to eradicate the latent reservoir of infected cells^3^ but have been unsuccessful except in one notable example^4^. In addition, substantial technological and financial hurdles preclude the widespread use of many developing cure strategies. The anti-proliferative therapies we propose here are used widely, permitting broad and immediate availability following a proof of efficacy study.

Several recent studies link cellular proliferation (both antigen-driven expansion and homeostatic proliferation) with persistence of the HIV reservoir on long-term ART (>1 year)^5–13^. Using a mathematical model, we demonstrate that continuous, modest reductions in latent cell proliferation rates would deplete the latent reservoir more rapidly than comparable increases in HIV activation as occurs with latency reversing agents. Further, we find that more rapid reservoir elimination on anti-proliferative therapy occurs with lower pre-treatment reservoir size and higher proportions of rapidly proliferating effector and central memory CD4^+^ T cells in the reservoir.

Based on analogies to finance, we call this strategy “compound interest cure”. We demonstrate the promise of the compound interest approach by identifying reservoir reduction commensurate with predictions from our model in HIV-infected patients treated with mycophenolate mofetil (MMF) in past studies. We confirm the anti-proliferative effect of MMF on naïve and memory CD4^+^ T cell subsets via *in vitro* experiments.

## Results

### ART decouples latent pool dynamics from ongoing infection

**Figure 1:**
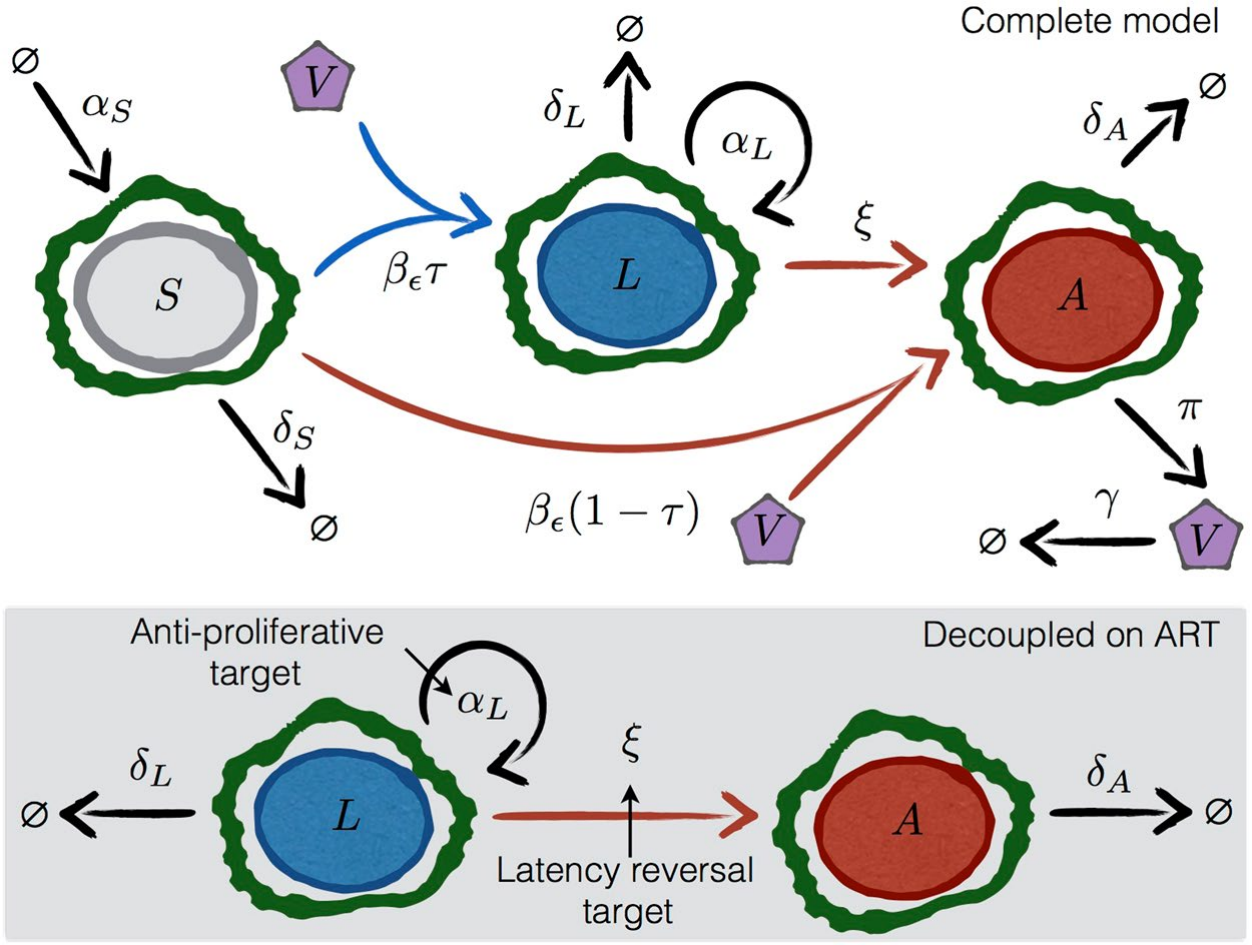
Schematics of models for HIV dynamics on and off ART. The top panel shows all possible transitions in the model (equation (1)). The bottom shaded panel shows the available transitions for the decoupled dynamic equations when ART suppresses the virus. Model parameters are given in Table 1. HIV virus *V* infects susceptible cells *S* at rate *β* reduced by ART of efficacy *ε* to *β*_*ε*_. The probability of latency given infection is *τ*. The rate of activation from latently infected cells (*L*) to actively infected cells (*A*) is *ξ*. Cellular proliferation and death are determined by rates *α* and *δ* for each compartment. The mechanisms of action of anti-proliferative and latency reversal therapies are to decrease *α*_*L*_ and increase *ξ*, respectively.

Our model is visualized in Fig. 1 and detailed in the Methods. If ART is perfectly effective, all susceptible cells are protected from new infection, even when cells activate from latency. Thus, the dynamics of the latent cells can be considered separately, decoupled from the dynamics of the other cell types, and the only mechanisms changing the latent cell pool size are cell proliferation, death, and activation (bottom panel, Fig. 1).

However, perfectly effective ART is not strictly necessary to consider the latent pool separately. As previously described^14, 15^, we define ART “critical efficacy” *ε*_*c*_ as the ART efficacy above which there is no set-point viral load, *i*.*e*. virus decreases rapidly with time (see Methods). Above the critical efficacy, viral production from activation could cause some new cell infection, but because the probability of latency (*τ*) is so low, new infection does not affect reservoir size or dynamics meaningfully. Using parameters from Table 1, we find *ε*_*c*_ ~ 85%. Because true ART efficacy is generally greater than this efficacy^16^, we predict little *de novo* infection in ART-suppressed patients, consistent with the lack of viral evolution following years of ART without re-seeding of the latent reservoir^8, 10, 11, 13, 17^.

**Table 1:** Parameters used in the HIV latency model.

Parameter	Value	Dimensions	Source	Meaning
*θ*_*L*_	−5.2 × 10^−4^	day^−1^	1, 26	net latent clearance rate on ART
*δ*_*L*_	0.0155	day^−1^	calculated*	latent central memory cell death rate
*α*_*L*_	0.015	day^−1^	49	latent cell proliferation rate
*α*_*cm*_	0.015	day^−1^	49	latent central memory cell Tcm proliferation rate
*α*_*em*_	0.047	day^−1^	49	latent effector memory cell Tem proliferation rate
*α*_*n*_	0.002	day^−1^	49	latent naïve cell Tn proliferation rate
*ξ*	5.7 × 10^−5^	day^−1^	21	activation rate
*α*_*A*_	0	day^−1^	45	active proliferation rate
*δ*_*A*_	1.0	day^−1^	50	active death rate
*τ*	10^−4^	day^−1^	29	probability of latency given infection
*α*_*S*_	300	cells/(*μ*L-day)	51	susceptible growth rate
*δ*_*S*_	0.2	day^−1^	51	susceptible death rate
*β*	10^−4^	*μ*L/(virus-day)	45, 51	HIV infectivity off ART
*β*_*ε*_	*β*(1 − *ε*)	*μ*L/(virus-day)	14, 15	HIV infectivity on ART (with efficacy $\varepsilon \in [0,1]$)
*π*	10^3^	virus/(cell-day)	45, 52	viral production rate
*γ*	23	day^−1^	53	viral clearance rate

All cellular rates are for CD4^+^ T cells. *Death rates for each cell type are calculated using the total clearance as *δ*_*i*_ = *α*_*i*_ − *ξ* − *θ*_*L*_ with $i\in [L,cm,em,n]$.

### Sustained mild effects on clearance rate deplete the reservoir more rapidly than large, one-time reservoir reductions

**Figure 2:**
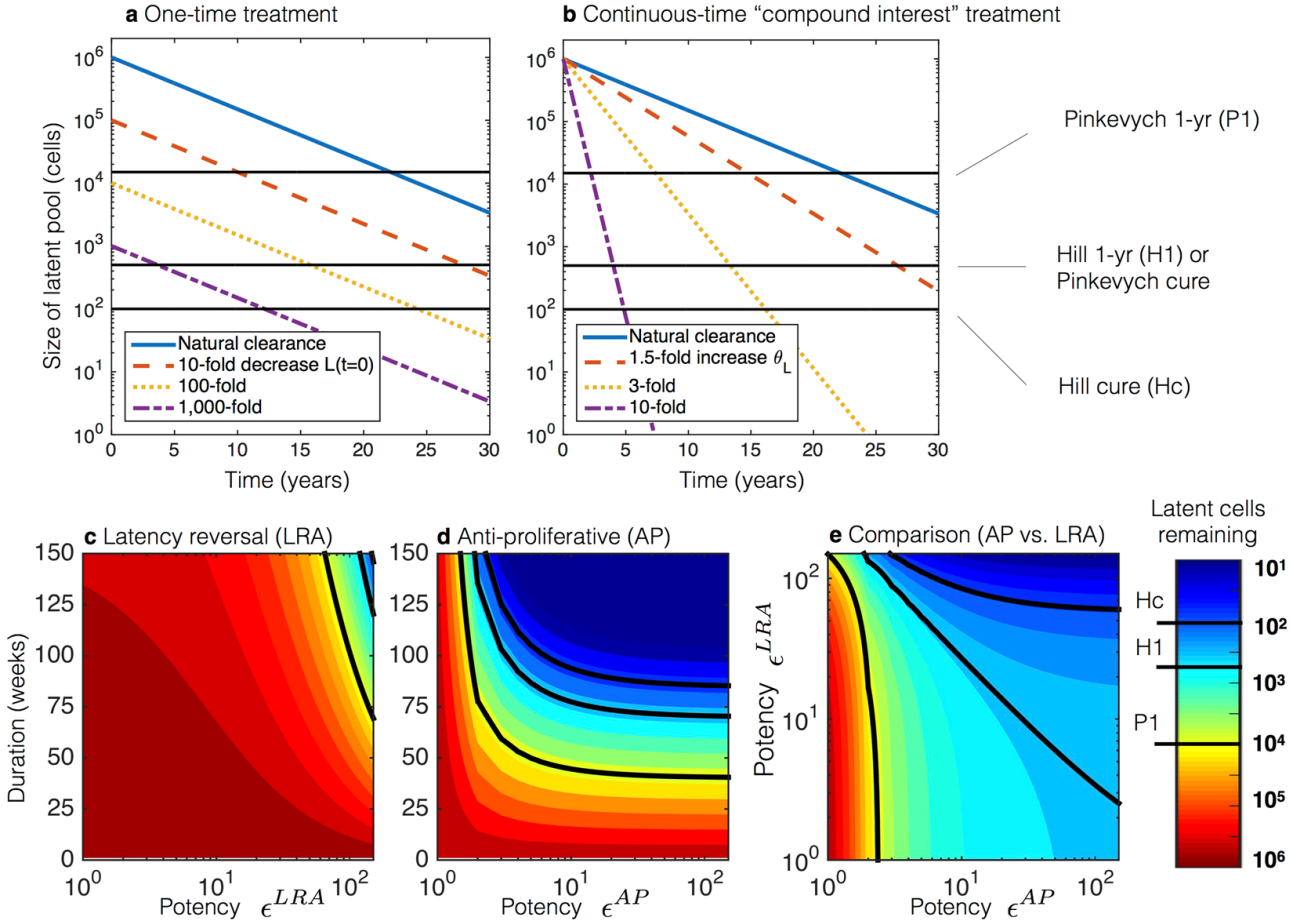
Simulated comparisons of latent reservoir eradication strategies on standard antiretroviral (ART) treatment. Treatment thresholds (discussed in Methods) are shown as solid black lines both in the plots and color bar, which is consistent between panels. (**a**) One-time therapeutic reductions of the latent pool (*L*_0_). (**b**) Continuous therapeutic increases in the clearance rate (*θ*_*L*_). Relatively small decreases in the clearance rate *θ*_*L*_ produce markedly faster times to cure than much larger decreases in the initial reservoir size. (**c**–**e**) Latency reversal agent (LRA) and anti-proliferative (AP) therapies are given continuously for durations of weeks with potencies given in fold increase in activation rate (*ε* ^*LRA*^) and fold decrease in proliferation rate (*ε* ^*AP*^), respectively. The color bar is consistent between panels, and thresholds of cure are shown as solid black lines both on plots and on the color bar. (**c**) Latency reversing agent therapy (LRA) administered alone requires years and potencies above 100 to achieve the cure thresholds. (**d**) Anti-proliferative therapies (AP) administered alone lead to cure thresholds in 1–2 years provided potency is greater than 2–3. (**e**) LRA and AP therapies are administered concurrently, and the reduction in the latent pool is measured at 70 weeks. Because the proliferation rate is naturally greater than the activation rate, increasing the AP potency has a much stronger effect than increasing the LRA potency.

The HIV cure strategy most extensively tested in humans is “shock-and-kill” therapy: latency reversing agents activate HIV in latent cells to replicate and express HIV proteins, allowing immune clearance while ART prevents further infection^3^. Other strategies in development include therapeutic vaccines^18^, viral delivery of DNA cleavage enzymes^19^, and transplantation of modified HIV-resistant cells^20^ informed by the “Berlin patient”^4^. Some of these therapies manifest as one-time reductions in the number of latent cells. We simulate such instantaneous decreases using equation (4) and cure thresholds described in Methods. Briefly, using ART interruption data, Hill *et al*. and Pinkevych *et al*. estimated the number of latently infected cells that would result in ART-free suppression of viremia for one year (Hill 1-yr and Pinkevych 1-yr) versus 30 years, Hill cure (Hc), in 50% of HIV-infected patients^21, 22^. With the reservoir clearance rate *θ*_*L*_ constant and a 100-fold reduction in reservoir size *L*_0_, the Pinkevych 1-yr threshold is immediately satisfied, but the Hill 1-yr and Pinkevych cure still require 15 years of ART. Hill cure requires a 1,000-fold reduction and more than 10 subsequent years of ART (Fig. 2a).

Continuous-time interventions are more promising. Relatively small changes in *θ*_*L*_ in equation (4) lead to significant changes in the time to cure (Fig. 2b). On ART alone, estimated cure occurs at roughly 70 years^1^. However, just a 3-fold increase in clearance rate achieves Hill cure in fewer than 20 years. A 10-fold sustained increase requires only five years for Hill cure.

Further, when continuous-time therapies are given, outcomes improve more by extending duration than by equivalent increases in potency (Fig. 2c,d demonstrate this given the substantial asymmetry of the contours over their *y* = *x* axes). Analogous to the so-called “miracle of compound interest,” increasing the clearance rate for an extended duration produces profound latency reduction.

### Smaller reductions in proliferation rate achieve more rapid reservoir depletion than comparable relative increases in activation rate

Latency reversing therapy can be modeled with equation (3) if treatment is assumed to be a continuous-time multiplication of activation. Simulations at various potencies and therapy durations indicate both Hill and Pinkevych cure thresholds require more than a 100-fold multiplication of *ξ* sustained for two or three years, respectively (Fig. 2c).

The latent cell proliferation rate is considerably larger than the activation rate (${\alpha }_{L}\gg \xi$, Table 1). Thus, anti-proliferative therapies would clear the reservoir faster than equivalently potent latency reversing strategies. When the reservoir of CD4^+^ T cells harboring replication-competent HIV is assumed to consist only of central memory cells (T_cm_), a 10-fold reduction in *α*_*cm*_ leads to Pinkevych 1-yr, Hill 1-yr, Pinkevych cure, and Hill cure in 0.8, 1.6, 1.6, and 1.8 years, respectively (Fig. 2d).

The improvement in cure time (when compared to an equivalent 10-fold increase in net reservoir clearance rate *θ*_*L*_) is possible because decreasing the proliferation rate means the net clearance rate approaches the latent cell death rate *δ*_*L*_. In fact, potency is relatively unimportant beyond reducing the proliferation rate by a factor of ten because the underlying death rate *δ*_*L*_ is the bound on clearance rate. The relative impact of anti-proliferative therapy is greater than that of latency reversing therapy when the two therapies are given concurrently for 70 weeks (Fig. 2e).

### Heterogeneity in reservoir cell types may necessitate prolonged anti-proliferative therapy

**Figure 3:**
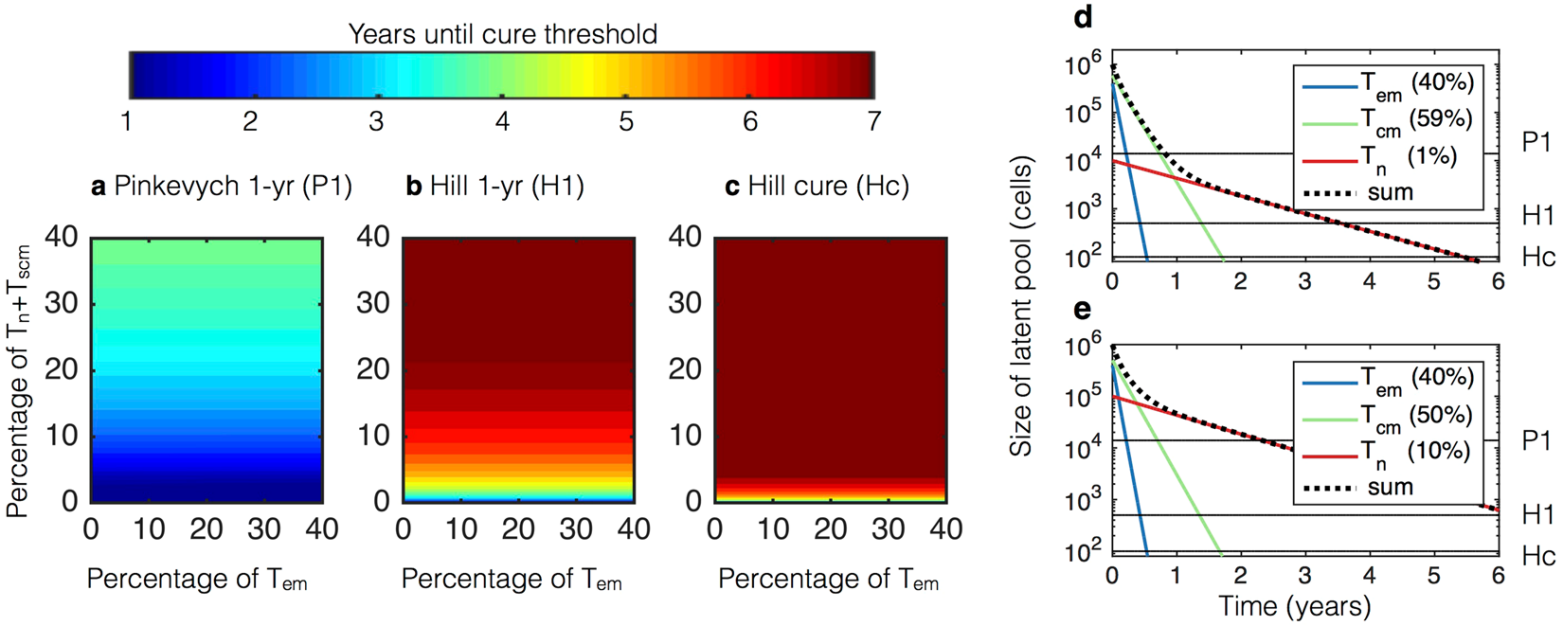
Simulated comparisons of anti-proliferative therapies on standard antiretroviral therapy (ART) assuming variable reservoir composition. Proliferation and death rates in Table 1. The potency of the therapy is *ε* ^*AP*^ = 10 (*i*.*e*., each cell type *i* has proliferation rate equal to *α*_*i*_/10 with $i\in [{\rm{em}},{\rm{cm}},{\rm{n}}]$). Plausible initial compositions of the reservoir (*L*_*i*_(0)) are taken from experimental measurements^6, 12, 23^. It is assumed that the HIV activation rate *ξ* is equivalent across all reservoir subsets. (**a**–**c**) Plots of times to therapeutic landmarks on long-term ART and anti-proliferative therapy with heterogeneous reservoir compositions consisting of effector memory (T_em_), central memory (T_cm_), and naïve plus stem cell-like memory (T_n_ + T_scm_) CD4^+^ T cells. T_em_ and T_n_ + T_scm_ percentages are shown with the remaining cells representing T_cm_. Times to one-year remission and functional cure are extremely sensitive to percentage of T_n_ + T_scm_ but not percentage of T_em_. (**d**,**e**) Continuous 10-fold therapeutic decreases in all proliferation rates (*α*_*i*_) result in Hill 1-yr in (**d**) 3.5 years assuming T_n_ + T_scm_ = 1% and (**e**) 6 years assuming T_n_ + T_scm_ = 10%. The reservoir is predicted to become T_n_ + T_scm_ dominant within 2 years under both assumptions, providing an indicator to gauge the success of anti-proliferative therapy in potential experiments.

Recent studies indicate that the reservoir is heterogeneous, consisting of CD4^+^ central memory (T_cm_), naïve (T_n_), effector memory (T_em_), and stem cell-like memory (T_scm_) T cells. Further, reservoir cell composition differs dramatically among patients^6, 12, 23^. This heterogeneity suggests the potential for variable responses to anti-proliferative agents. Proliferation rates of T_cm_ (once per 66 days) exceed T_n_ (once every 500 days) but lag behind T_em_ proliferation rates (once every 21 days, Table 1). In our model T_scm_ are assumed to proliferate at the same frequency as T_n_ based on similar properties. We simulate possible reservoir profiles with different percentages of T_n_, T_cm_, and T_em_ in Fig. 3a–c. At least 7 years of treatment is needed for Pinkevych functional cure (Hill 1-yr) if slowly proliferating cells (T_n_ and/or T_scm_) comprise more than 20% of the reservoir. In contrast, an increased proportion of T_em_ has no clinically meaningful impact on time to cure. Slowly proliferating cells are predicted to comprise the entirety of the reservoir within two years of 10-fold anti-proliferative treatment regardless of initial percentage of T_n_ or T_scm_ (Fig. 3d,e).

The uncertainty in the reservoir composition tempers the results in Fig. 2. On the other hand, our model assumes that the HIV activation rate *ξ* is equivalent across all CD4^+^ T cell reservoir subsets. It is biologically plausible, though unproven, that latent cell proliferation and activation are linked processes and that therefore HIV rarely or never activates from resting T_n_ or T_scm_. Under this assumption, functional cure might occur once T_em_ and T_cm_ reservoirs have been reduced to the Hill cure level, *i*.*e*. approximately 1.5 years in Fig. 3d,e.

### Initial reservoir size, anti-proliferative potency, and reservoir cell subtypes predict time to cure

**Figure 4:**
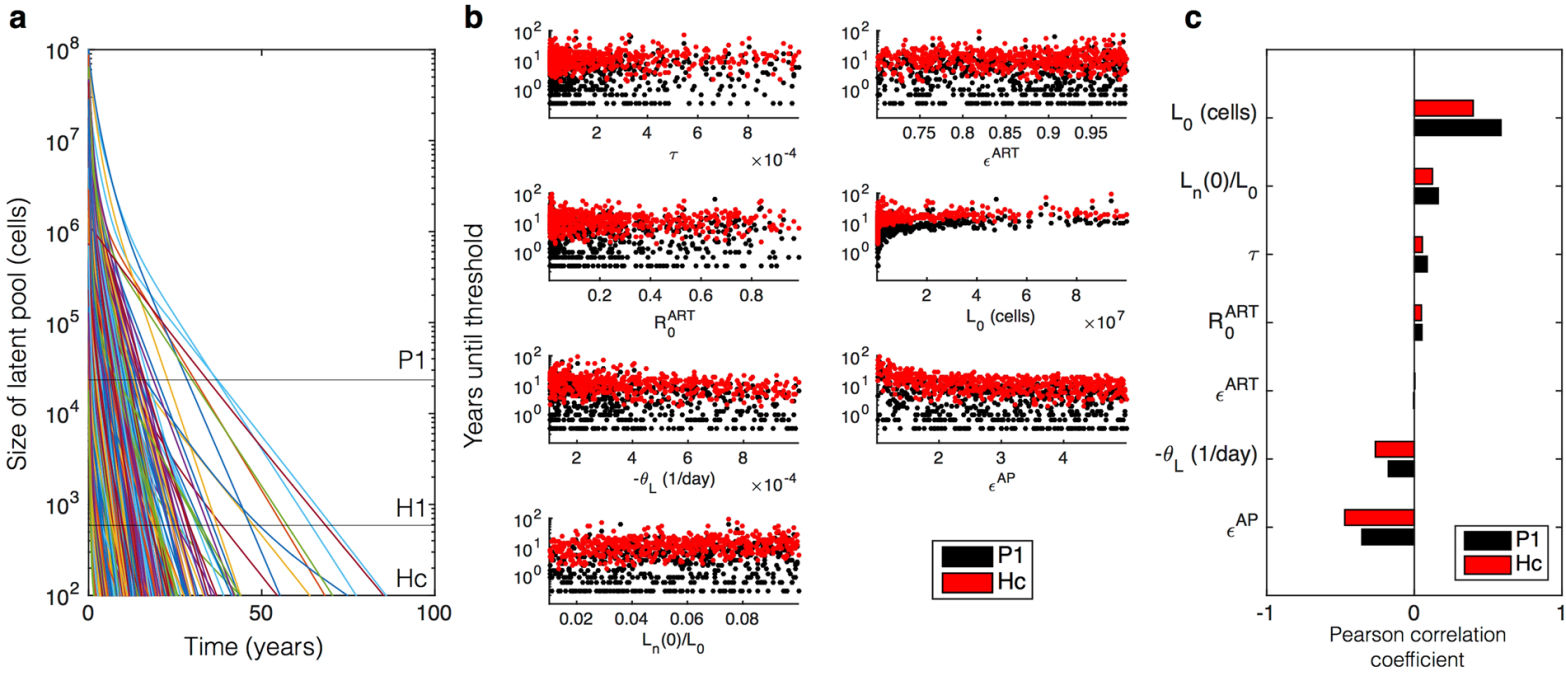
Global sensitivity analysis. We use the ranges of parameters from Supplementary Table S4. (**a**) 1,000 simulations drawn from Latin Hypercube sample parameter sets where ${R}_{0}^{ART} < 1$ are shown to demonstrate the variability of latent pool dynamics with respect to all combinations of parameter ranges. (**b**) The time until each cure threshold, Pinkevych 1-yr (P1) and Hill cure (Hc), are calculated as the time when the latent reservoir contains fewer than 20,000 and 200 cells respectively. In some cases cures are achieved within months. In others, cure requires many years. (**c**) Pearson correlation coefficients indicate the correlations between each variable and time to cure. *L*_0_ is the initial number of latent cells. *L*_*n*_(0)/*L*_0_ is the initial fraction of naïve cells in the latent pool. *τ* is the probability of latency given infection. ${R}_{0}^{ART}$ is the basic reproductive number on ART. *ε* ^*ART*^ is the percent decrease in viral infectivity in the presence of ART. *θ*_*L*_ is the decay rate of latent cells. *ε* ^*AP*^ is the fold reduction in proliferation rate.

**Figure 5:**
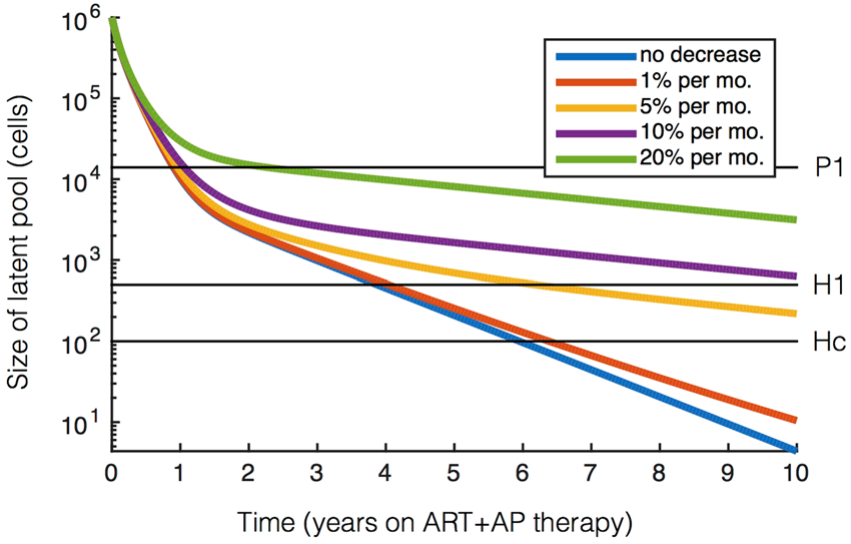
Waning anti-proliferative potency over-time modulates cure. Latent reservoir dynamics on combined ART and anti-proliferative therapy simulated for waning potency of anti-proliferative therapy over time. The latent reservoir size is shown with horizontal black lines corresponding to the cure threshholds used throughout the paper. Cure thresholds are achieved within 10 years if potency decreases by less than 5% per month considering 1% naïve T cells (*L*_*n*_(0)/*L*_0_ = 0.01) and initial anti-proliferative potency *ε* ^*AP*^ = 5.

Using literature-derived ranges for the parameters of interest, we completed a global sensitivity analysis to examine which factors might impact time to cure in a heterogeneous patient pool developed by Latin Hypercube sampling of a broad parameter space^24^ (Fig. 4). We correlate variables with time to cure on ART/anti-proliferative combination therapy. Varying the probability of latency given infection (*τ*) does not change time to cure. Similarly, varying the basic reproductive number on ART (${R}_{0}^{ART}$), a measure of ART efficacy, defined as the number of new infected cells generated by one infected cell during ART, does not change time to cure. On the other hand, as the pre-treatment size of the latent pool *L*_0_ increases, the necessary time to cure also increases. Increasing anti-proliferative therapy potency *ε* ^*AP*^ decreases cure time. Increasing percentages of naïve T cells *L*_*n*_(0)/*L*_0_ in the latent reservoir delay the time to cure while a faster latent decay rate *θ*_*L*_ hastens cure. Finally, we simulated the possibility of a diminishing impact of anti-proliferative therapy over time in Fig. 5. The simulation shows that when potency decreases by less than 5% per month, cure thresholds are still achieved within 10 years of ART and anti-proliferative treatment. The fastest waning of potency (20% per month) results in return to the natural clearance rate within the first 2 years of therapy prompting longer times to cure.

### Model output is congruent with available clinical data

Chapuis *et al*. treated eight ART-suppressed, HIV-infected patients with 24 weeks of mycophenolate mofetil, a licensed anti-proliferative agent. As a marker of anti-proliferative effect, the percentages of Ki67^+^ CD4^+^ T cells were measured before and after MMF treatment (2 × 500 mg daily) and were found to have decreased on average 2.2-fold. Incorporating that reduction in latent cell proliferation rate *ε* ^*AP*^ = 2.2 over 24 weeks of treatment, we estimate a 10- to 40-fold reduction in the latent reservoir (see Fig. 2d). Chapuis *et al*. found a 10- to 100-fold reduction in infectious units per million (IUPM) by quantitative viral outgrowth assay in five patients, comparable to our estimate^25^. These reductions far exceed natural reservoir clearance rates and are consistent with a therapeutic effect^26^.

García *et al*. assessed the effect of MMF (2 × 250 mg daily) on HIV in the context of ART treatment interruption^27^. Seventeen HIV-infected patients received ART for a year and then were randomized into a control group that remained on ART only and an experimental group that also received MMF for 17 weeks. ART was interrupted in both groups and viral rebound assessed. MMF inhibited CD4^+^ T cell proliferation (as measured by an *in vitro* assay) in six of nine MMF recipients (responders). The time to rebound was 1–4 weeks in the control group and 6–12 weeks in the MMF-responder group. Using results from Pinkevych *et al*., a median time to rebound of seven weeks (see Fig. 5b of ref. 22) corresponds to a 7-fold decrease in the latent reservoir. Using results from Hill *et al*., the same median time to detection of seven weeks (see Fig. 4 of ref. 21) corresponds to a 50-fold reduction in the latent reservoir. These calculations are congruent with our model’s estimate that 17 weeks of MMF treatment at potency *ε* ^*AP*^ = 2.2 leads to a 10-fold reduction in the reservoir.

### MMF decreases proliferation in CEM cells, CD4^+^ T cells from HIV positive and negative donors, and all CD4^+^ T cell subsets

**Figure 6:**
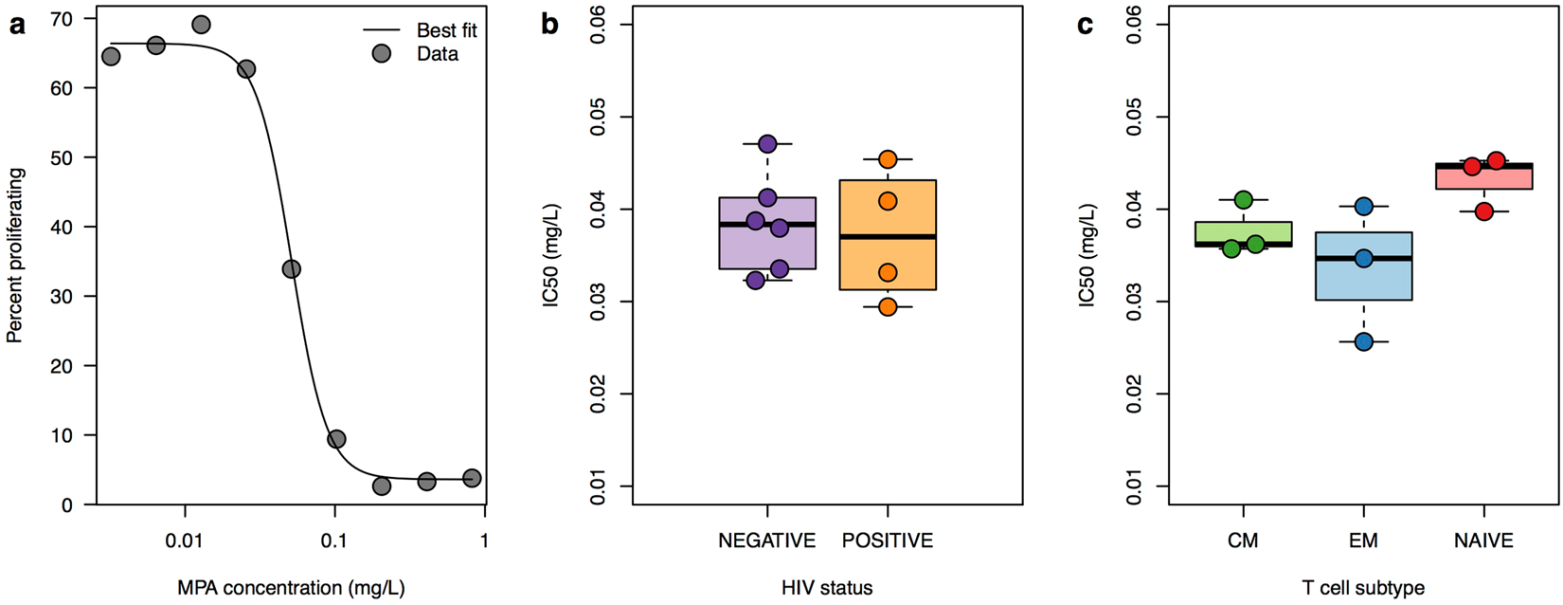
MMF pharmacodynamics. Pure mycophenolic acid (MPA) was added to CEM cells at varying concentrations and proliferation of CEM cells was measured to determine a dose-response curve and Hill slope. CD4^+^ T cells from stored peripheral blood mononuclear cell samples from 10 participants (4 HIV-infected, 6 HIV-uninfected) were stimulated to proliferate. CD4^+^ T cells from 3 HIV-negative subjects were sorted into effector memory (EM), central memory (CM), and naïve subsets. Pure MPA was added to these cells at varying concentrations in order to determine IC50s for MPA. (**a**) Dose-response curve with percentages of CEM cells proliferating at varying doses of MPA. The Hill slope is −3.7. (**b**) 4 samples from HIV-positive participants and 6 samples from HIV-negative participants had similar IC50s. (**c**) IC50s were similar among CD4^+^ effector memory (EM), central memory (CM), and naïve T cell subsets.

To explain the heterogeneous impact of MMF treatment (three of six in Chapuis *et al*. did not demonstrate a meaningful reservoir clearance; three of nine patients in García *et al*. had a weak anti-proliferative response to MMF and no delay in HIV rebound upon ART cessation), we conducted an *in vitro* study of MMF pharmacodynamics. We titrated the capacity of mycophenolic acid to inhibit spontaneous proliferation of cells from a human T lymphoblastoid cell line (CEM cells)^28^ and identified a steep Hill slope of −3.7 (Fig. 6a). A Hill slope with absolute value greater than one indicates cooperative binding at the site of drug action and implies a sharp transition from negligible to complete therapeutic effect at a specific drug concentration. These results explain how patients with inadequate MMF dosage could have a limited anti-proliferative effect.

We tested the capacity of mycophenolic acid (MPA), the active metabolite of MMF, to inhibit CD4^+^ T cell proliferation in CD4^+^ T cells from four HIV-positive and six HIV-negative participants and found similar IC50s (Fig. 6b). Further, CD4^+^ T cells from three HIV-negative participants were sorted into central-memory, effector-memory, and naïve subsets. Similar proliferation inhibition was observed in all three cell subsets (Fig. 6c). These results suggest a potential for MMF to deplete the HIV reservoir.

## Discussion

We developed a mathematical model of HIV dynamics to study various cure strategies^21, 29^. We demonstrate that minor reductions in CD4^+^ T cell proliferation rates would exhibit powerful reductions in the latent reservoir when therapy duration is extended over time. We call this proposed strategy “compound interest cure” due to the correspondence with financial modeling.

Our results are relevant because the HIV cure strategy most rigorously being tested in humans—latency reversal therapy (“shock-and-kill”)—may not capitalize on the advantages of a compound interest approach. Promising latency reversing agents are typically dosed over short time-frames due to concerns about toxicity. T cell activation does not always lead to induction of HIV replication providing another potential limitation of latency reversing therapy^30^. Furthermore, even if these agents exert a large relative impact on the activation rate of memory CD4^+^ T cells, we predict the reduction in the reservoir may be insignificant given that the natural activation rate is orders of magnitude lower than proliferation and death rates. Latency reversal agents are also being considered in conjunction with other interventions such as engineered antibodies and/or T cells. These combined approaches carry additional unknown toxicities and rely on the effectiveness of latency reversal agents. Most challenging of all, these experimental therapies could be prohibitively expensive to implement globally.

The theoretical potential of the anti-proliferative approach is worthy of a clinical trial given the existence of licensed medications that limit T cell proliferation, including MMF. In line with our prediction that duration is more important than potency, these drugs are dosed over months to years for rheumatologic diseases and preventing rejection after solid organ transplant. The most frequent side effects reported are gastrointestinal symptoms and increased risk of infection though the latter risk is obscured by concurrent use of high-dose glucocorticoids^31^. MMF has been given to several hundred HIV-infected patients suppressed on ART^25, 27, 32–40^ (reviewed in Supplementary information). In this population, neither opportunistic infections nor adverse events were increased, and CD4^+^ T cell counts did not decrease significantly during therapy. We hypothesize that whereas MMF decreases proliferation of existing CD4^+^ T cells, it does not suppress thymic replenishment of these cells. Finally, MMF did not counteract the effects of ART^25, 27^, and we do not expect viral drug resistance or ongoing viral evolution to occur on anti-proliferative therapy. Despite these reassuring findings, future studies of HIV-infected patients on anti-proliferative agents will require extremely close monitoring for drug toxicity and immunosuppression. In addition, mycophenolic acid has a large Hill coefficient, suggesting a narrow therapeutic range. We suspect that the participants who did not respond to MMF in the clinical studies described above^25, 27^ required higher drug concentrations.

Our model suggests that slowly proliferating cells in the reservoir could present a barrier to rapid eradication of latently HIV-infected cells. Therefore, anti-proliferative strategies may face a challenge akin to the cancer stem cell paradox, whereby only the rapidly proliferating tumor cells are quickly expunged with chemotherapy. For example, tyrosine kinase inhibitors suppress proliferation of cancer cells in chronic myelogenous leukemia (CML). While many patients achieve “undetectable minimal residual disease,” some patients relapse to pre-therapy levels of disease following therapy cessation—perhaps due to slowly proliferating residual cancer stem cells^41^. Additional limitations could include insufficient anti-proliferative drug delivery to anatomic sanctuaries, certain cellular subsets that are unaffected by treatment, and cytokine-driven feedback mechanisms that compensate for decreased proliferation by increasing memory CD4^+^ T cell lifespan. These challenges might be countered by combining anti-proliferative agents with other cure therapies. Avoidance of nucleoside and nucleotide reverse transcriptase inhibitors, which may enhance T cell proliferation, could provide an important adjunctive benefit^42, 43^.

The anti-proliferative approach is attractive because it is readily testable without the considerable research and development expenditures required for other HIV cure strategies. Anti-proliferative approaches require minimal potency relative to latency reversing agents, and T cell anti-proliferative medications are well studied mainstays of organ rejection prevention. Therefore, we propose trials with anti-proliferative agents as an important next step in the HIV cure agenda.

## Methods

### Latent reservoir dynamic model

We based our model (schematic in Fig. 1) on previous HIV dynamics models^29, 44^. We follow the concentrations [cells/*μ*L] of susceptible CD4^+^ T cells *S*, latently infected cells *L*, actively infected cells *A*, and plasma viral load *V* [copies/*μ*L] over time. The system of ordinary differential equations (using the over-dot to denote derivative in time)

1 $$\begin{array}{rcl}\dot{S} & = & {\alpha }_{S}-{\delta }_{S}S-{\beta }_{\varepsilon }SV\\ \dot{L} & = & {\alpha }_{L}L+\tau {\beta }_{\varepsilon }SV-{\delta }_{L}L-\xi L\\ \dot{A} & = & \mathrm{(1}-\tau ){\beta }_{\varepsilon }SV-{\delta }_{A}A+\xi L\\ \dot{V} & = & \pi A-\gamma V\end{array}$$

tracks these state variables. We define *α*_*S*_ [cells/*μ*L-day] as the constant growth rate of susceptible cells, *δ*_*S*_ [1/day] as the death rate of susceptible cells, and *β*_*ε*_ = (1 − *ε*)*β* [*μ*L/virus-day] as the therapy-dependent infectivity. We define *ε* [unitless] as the ART efficacy, ranging from 0 (meaning no therapy) to 1 (meaning perfect therapy). *α*_*L*_ and *δ*_*L*_ [1/day] are the proliferation and death rates of latent cells, respectively. The death rate of actively infected cells is *δ*_*A*_, and the proliferation rate of activated cells ${\alpha }_{A}\approx 0$ is likely negligible^45^. *τ* [unitless] is the probability of latency given infection, and *ξ* [1/day] is the rate of transition from latent to actively infected cells. The viral production rate is *π* [virions/cell-day], which describes the aggregate rate of constant viral leakage and burst upon cell death. *γ* [1/day] is the HIV clearance rate. Parameter values are given in Table 1.

Additional calculations including derivations of equilibrium solutions and stability analysis as well as further discussion of model parameter derivations are presented in the Supplementary information.

### The compound interest formula

In the Supplementary information, we determine the critical drug efficacy *ε*_*c*_, the value of *ε* above which viral load quickly decays. Moreover, when *ε* > *ε*_*c*_, we can consider the latent cell equation in isolation:

2 $$\dot{L}={\alpha }_{L}L-{\delta }_{L}L-\xi L.$$

Defining the initial number of latent cells as *L*_0_ gives

3 $$L={L}_{0}{e}^{({\alpha }_{L}-{\delta }_{L}-\xi )t}.$$

Equation (3) implies that the clearance rate of latently infected cells is a function of their proliferation, death, and activation rates. Defining the total clearance rate *θ*_*L*_ = *α*_*L*_ − *δ*_*L*_ − *ξ*, we see a mathematical correspondence to the principle of continuous compound interest with *L*_0_ as the principal investment and *θ*_*L*_ as the interest rate:

4 $$L={L}_{0}{e}^{{\theta }_{L}t}.$$

Experimental measurements indicate an average latent cell half-life of 44 months (*θ*_*L*_ = −5.2 × 10^−4^ per day)^1, 26^ and an average latent reservoir size *L*_0_ of one-million cells^1^. Note that when *θ*_*L*_ < 0, the latent reservoir is cleared exponentially. Alternatively, if *α*_*L*_ exceeds the sum of *ξ* and *δ*_*L*_, *L* grows indefinitely.

### Composition of the latent reservoir: modeling T cell subsets

We include heterogeneity in T cell phenotype into the model by splitting the differential equation for the latent cells into three differential equations, one for each subtype *L*_*i*_ with $i\in [cm,em,n]$. We ignore transitions between phenotype because the composition of the reservoir is reasonably stable over time^12^. Our extended model is the system

5 $${\dot{L}}_{i}={\theta }_{i}{L}_{i}.$$

The total number of latent cells is the sum of the subset populations, $L={\sum }_{i}\,{L}_{i}$, and solution is

6 $$L(t)=\sum _{i}\,{L}_{i}\mathrm{(0)}{e}^{{\theta }_{i}t}$$

where *θ*_*i*_ = *α*_*i*_ − *δ*_*i*_ − *ξ*, and *L*_*i*_(0) are the initial numbers of each subtype.

Simulations assume the same net clearance rate and activation rates among subsets, but different proliferation rates *α*_*i*_ and different calculated death rates *δ*_*i*_ = *α*_*i*_ − *θ*_*L*_ − *ξ*. The initial conditions for each subtype *L*_*i*_(0) are inclusive of several varying measurements in the literature^6, 12, 23^. We consider Ttm to have the same proliferation rates as Tcm. Similarly, we characterize stem-cell-like memory CD4^+^ T cells (Tscm) as Tn given their slow turnover rate. Of note, these are conservative estimates that would not favor anti-proliferative therapy. In Fig. 5, we allow the anti-proliferative potency to decrease over time by assuming ${\alpha }_{i}(t)={\alpha }_{i}[1+({\varepsilon }^{AP}-1)\,\exp (-\varphi t)]$ for each T cell subset in equation 5. Here *ϕ* is the waning potency rate that ranges from 0–20% per month. We assume the initial potency is a 5-fold decrease *ε* ^*AP*^ = 5, and we use a 1 million cell reservoir having 1% naïve T cells. We solve the equation for each subset numerically with ${\mathtt{ode23s}}$ in Matlab, summing the subset dynamics after solving.

### Reservoir reduction targets for cure strategies

We use experimentally derived thresholds to compare potential cure therapies in the framework of our model. Hill *et al*. employed a stochastic model to estimate that a 2,000-fold reduction in the latent pool would result in HIV suppression off ART for a median of one year. After a 10,000-fold reduction in latent cells, 50% of patients would remain functionally cured (ART-free remission for at least 30 years)^21^. Pinkevych *et al*. inferred from analytic treatment interruption data that decreasing the latent reservoir 50–70-fold would lead to HIV remission in 50% of patients for one year^22^. Using the Pinkevych *et al*. results, we extrapolate a functional cure threshold as a 2,500-fold decrease in the reservoir size (Supplementary information). Given ongoing debate in the field, we consider all four thresholds—henceforth referred to as Hill 1-yr, Hill cure, Pinkevych 1-yr, and Pinkevych cure.

### Sensitivity analysis

To examine the full range of possible outcomes we completed a global sensitivity analysis of the model in which all variables were simultaneously varied in the ranges of Table S5 by logarithmically covering Latin Hypercube sampling^24^. The simulations were carried out in Matlab using ${\mathtt{lhsdesign}}$ and ${\mathtt{ode23s}}$. We correlated each parameter of interest with the time to reach the Hill and Pinkevych cure thresholds. Calling the time-to-cure *T*, correlations were calculated with the Pearson correlation coefficient: the covariance of *T* with each parameter of interest *p* normalized by both the standard deviation of *T* and that of *p*, that is $\rho ={\rm{cov}}(T,p)/{\sigma }_{T}{\sigma }_{p}$. 1,000 simulations were carried out, keeping only the parameter combinations leading to reservoir decay, i.e. those satisfying ${R}_{0}^{ART} < 1$.

### Mycophenolic acid anti-proliferation assay methods

Blood samples for the MPA *in vitro* studies were obtained from ART-treated, HIV-infected and healthy, HIV-negative men at the HIV Vaccine Trials Unit Clinic in Seattle, Washington. All procedures were approved by the Institutional Review Boards of the University of Washington and the Fred Hutchinson Cancer Research Center (IRB 1830 and 5567) and were performed in accordance with institutional guidelines and regulations. Written informed consent was obtained from each donor.

Cells were labeled using the CellTrace Violet Cell Proliferation Kit (Invitrogen) by incubation in 40 *μ*M CellTrace Violet in Roswell Park Memorial Institute (RPMI) cell culture media with penicillin/streptomycin and L-glutamine (Gibco) plus 10% fetal bovine serum (Gemini Bio-Products) (R-10 media) for five minutes at room temperature^46^ followed by washing twice with R-10. Peripheral blood mononuclear cells were stimulated with 1 *μ*g/mL staphylococcal enterotoxin B (SEB; Sigma-Aldrich) and 10 IU/mL IL-2 (Peprotech). Sorted CD4^+^ T cell subsets (naïve, effector memory, and central memory) were stimulated with Dynabeads Human T-Activator CD3/CD28 beads (Gibco) at a bead to cell ratio of 1:1 with 10 IU/mL IL-2. CEM cells were not stimulated, as they proliferate continuously. Pure mycophenolic acid (Sigma-Aldrich), the active metabolite of MMF, was added at concentrations ranging from 0.01 to 2.56 *μ*M. Cells were cultured in R-10 for 72 h.

After the culture period, cells were washed and stained with Fixable Live/Dead Yellow (Invitrogen), followed by CD45RA FITC, CD4 PE-Cy5, CCR7 BV785 (all BD), and CD3 ECD (Beckman Coulter) at the minimum saturating doses. Cells were then fixed with 1% paraformaldehyde and acquired on a five-laser BD LSRII flow cytometer (355, 405, 488, 535, and 633 nm). Live, single CD4^+^ T cells were gated into “proliferated” or “not proliferated” on the basis of CellTrace Violet fluorescence.

The IC50s and Hill slope were calculated using the ${\mathtt{drc}}$ package in ${\mathtt{R}}$ (Supplementary information)^47, 48^.

## Electronic supplementary material

Supplementary information
