# Supplementary material for: Anti-proliferative therapy for HIV cure: a compound interest approach
Source: Sci Rep. 2017 Jun 21;7:4011. doi: 10.1038/s41598-017-04160-3 (PMC5479830; doi:10.1038/s41598-017-04160-3)
Supplement: Supplementary file 1 — Supplementary information [file 41598_2017_4160_MOESM1_ESM.pdf]

# SUPPLEMENTARY INFORMATION ONLINE

## Anti-proliferative therapy for HIV cure: a compound interest approach

Daniel B Reeves, PhD[1◇], Elizabeth R Duke, MD[1,2◇], Sean M Hughes, MA[4], Martin Prlic, PhD[1,3], Florian Hladik, MD/PhD[1,2,4‡*], Joshua T Schiffer, MD[1,2,5‡*]

**1** Vaccine and Infectious Diseases Division, Fred Hutchinson Cancer Research Center, 1100 Eastlake Ave, Seattle, WA 98109 USA
**2** Department of Medicine, University of Washington, 1959 NE Pacific St, Seattle, WA 98105 USA
**3** Department of Global Health, University of Washington, 1959 NE Pacific St, Seattle, WA 98195 USA
**4** Departments of Obstetrics and Gynecology, University of Washington, 1959 NE Pacific St, Seattle, WA 98195 USA
**5** Clinical Research Division, Fred Hutchinson Cancer Research Center, 1100 Eastlake Ave, Seattle, WA 98109 USA

◇ These authors contributed equally to this work. ‡ These authors also contributed equally to this work.

Corresponding Authors: *jschiffe@fredhutch.org, *fhladik@fredhutch.org

# Contents

**1 Modeling methods** **2**
1.1 Equilibrium solutions leading to decoupled equations for latently infected cells. . . . . . . . . . . . . 2
1.2 Stability analysis of equilibrium solutions: Calculating the basic reproductive number confirms that decoupling latent cell equations is valid when ART efficacy is above the critical value. . . . . . . . . 2
1.3 Deriving the basic reproductive number $R_0$ with the next-generation matrix method . . . . . . . . . . 5
1.4 Allowing direct active to latent transitions has a neglible impact on reservoir size. . . . . . . . . . . . 7
1.5 Impact of duration and rates on clearance time . . . . . . . . . . . . . . . . . . . . . . . . . . . . 7

**2 Model parameters** **7**
2.1 Latent cell parameters: $\theta_L$, $\alpha_L$, $\delta_L$, $\xi$ . . . . . . . . . . . . . . . . . . . . . . . . . . . . 7
2.2 Susceptible and active cell parameters: $\alpha_S$, $\delta_S$, $\alpha_A$, $\delta_A$ . . . . . . . . . . . . . . . . . . . . . . 8
2.3 Estimating the infectivity $\beta$ . . . . . . . . . . . . . . . . . . . . . . . . . . . . . . . . . . . . 9
2.4 Viral parameters: $\pi$, $\gamma$ . . . . . . . . . . . . . . . . . . . . . . . . . . . . . . . . . . . . . 9
2.5 The latency fraction: $\tau$ . . . . . . . . . . . . . . . . . . . . . . . . . . . . . . . . . . . . . 9
2.6 Parameter estimates, ranges, and confidence intervals . . . . . . . . . . . . . . . . . . . . . . . 9

**3 Mycophenolate mofetil (MMF)** **10**
3.1 Determining the Hill coefficient and IC50s . . . . . . . . . . . . . . . . . . . . . . . . . . . . 10
3.2 Previous studies in HIV-infected patients treated with ART and MMF . . . . . . . . . . . . . . . . 10

# 1 Modeling methods

## 1.1 Equilibrium solutions leading to decoupled equations for latently infected cells.

Our model for HIV including latently infected cells and antiretroviral therapy (ART) is described completely in the main body with all rates specified in Table 1. Here we present the ordinary differential equations (ODEs) without further clarification:

$$\begin{aligned}
\dot{S} &= \alpha_S - \delta_S S - \beta_\epsilon S V \\
\dot{L} &= \theta_L L + \tau \beta_\epsilon S V \\
\dot{A} &= (1-\tau)\beta_\epsilon S V - \delta_A A + \xi L \\
\dot{V} &= \pi A - \gamma V
\end{aligned} \tag{S1}$$

Equilibrium solutions (denoted by the asterisk) can be calculated by setting $\dot{S}, \dot{L}, \dot{A}, \dot{V} = 0$. The system has two equilibrium solutions:

1. "Viral-free equilibrium", the values at which the system rests prior to infection

$$\begin{aligned}
S^* &= \alpha_S/\delta_S \\
L^* &= 0 \\
A^* &= 0 \\
V^* &= 0,
\end{aligned} \tag{S2}$$

2. "Setpoint equilibrium", the values at which the system has durable viral infection: commonly referred to as "viral setpoint" in clinical HIV care.

$$\begin{aligned}
S^* &= \frac{\gamma \delta_A}{\beta_\epsilon \pi \bar{\ell}} \\
L^* &= \frac{\tau}{\theta_L}\left[\frac{\gamma \delta_S \delta_A}{\beta_\epsilon \pi \bar{\ell}} - \alpha_S\right] \\
A^* &= \frac{\alpha_S \bar{\ell}}{\delta_A} - \frac{\gamma \delta_S}{\beta_\epsilon \pi} \\
V^* &= \frac{\alpha_S \pi \bar{\ell}}{\gamma \delta_A} - \frac{\delta_S}{\beta_\epsilon},
\end{aligned} \tag{S3}$$

where $\bar{\ell} = 1 - (1 + \xi/\theta_L)\tau$. We use the $\bar{\ell}$ notation to simplify the appearance of the equations and choose the lower-case $\bar{\ell}$, as this factor encapsulates all the latent dynamics. For $\tau \ll 1$, we have $\bar{\ell} \sim 1$, and $\tau$ is far less than one in the literature (see Table S4).

## 1.2 Stability analysis of equilibrium solutions: Calculating the basic reproductive number confirms that decoupling latent cell equations is valid when ART efficacy is above the critical value.

By linearizing our system of equations, we address the local stability of the equilibrium points. We begin by defining our state variables as a vector $\mathbf{x} = [S, L, A, V]^T$ such that we can express the system of ODEs as $\mathbf{F}(\mathbf{x}) = \partial_t \mathbf{x}$. Then, we Taylor expand this function around the equilibrium point $\mathbf{x}^*$ where $\mathbf{F}(\mathbf{x}^*) = 0$:

$$\mathbf{F}(\mathbf{x}) \approx \mathbf{F}(\mathbf{x}^*) + \left.\frac{\partial \mathbf{F}}{\partial \mathbf{x}}\right|_{\mathbf{x}^*} (\mathbf{x} - \mathbf{x}^*) + \left.\frac{\partial^2 \mathbf{F}}{\partial \mathbf{x}^2}\right|_{\mathbf{x}^*} (\mathbf{x} - \mathbf{x}^*)^2 + \ldots \tag{S4}$$

By construction the first term in the expansion is zero. Calling a small $\Delta \mathbf{x} \ll 1 = \mathbf{x} - \mathbf{x}^*$, we can neglect terms of $\mathcal{O}(\Delta \mathbf{x}^2)$ and higher. Using $\mathbf{x} = \mathbf{x}^* + \Delta \mathbf{x}$ we can rewrite

$$\mathbf{F}(\mathbf{x}) = \frac{d\mathbf{x}}{dt} = \frac{d\mathbf{x}^*}{dt} + \frac{d\Delta \mathbf{x}}{dt} \tag{S5}$$

and because $\mathbf{x}^*$ is not a function of time we are left with the linear equation we desire:

$$\frac{d}{dt}\Delta \mathbf{x} \approx \left.\frac{\partial \mathbf{F}}{\partial \mathbf{x}}\right|_{\mathbf{x}^*} \Delta \mathbf{x}. \tag{S6}$$

The matrix $\mathbf{J} = \partial_{\mathbf{x}} \mathbf{F}$ is referred to as the Jacobian matrix for our model, and is in complete form:

$$\mathbf{J} = \begin{bmatrix} -\delta_S - \beta_\epsilon V & 0 & 0 & \beta_\epsilon S \\ \tau \beta_\epsilon V & \theta_L & 0 & \tau \beta_\epsilon S \\ (1-\tau)\beta_\epsilon V & \xi & -\delta_A & (1-\tau)\beta_\epsilon S \\ 0 & 0 & \pi & -\gamma \end{bmatrix} \tag{S7}$$

We can evaluate the Jacobian at both equilibria derived above (Eq. S2 and Eq. S3). The eigenvalues of the Jacobian then govern how perturbations near equilibrium behave. If all eigenvalues $\lambda_j$ of the Jacobian have negative real components $Re(\lambda_j) < 0$, perturbations decay back to equilibrium and the equilibrium is deemed stable.[1] Before infection, we input the equilibrium solutions (Eq. S2) and the Jacobian of the viral free equilibrium (subscript vfe) is

$$\mathbf{J}_{\text{vfe}} = \begin{bmatrix} -\delta_S & 0 & 0 & \beta_\epsilon \alpha_S / \delta_S \\ 0 & \theta_L & 0 & \tau \beta_\epsilon \alpha_S / \delta_S \\ 0 & \xi & -\delta_A & (1-\tau)\beta_\epsilon \alpha_S / \delta_S \\ 0 & 0 & \pi & -\gamma \end{bmatrix} \tag{S8}$$

We can calculate the eigenvalues of this rather sparse matrix. Taking the determinant of $\mathbf{J} - \lambda \mathbb{1}_{4\times 4}$ and setting this equal to zero we find

$$0 = (-\delta_S - \lambda)\left\{(\theta_L - \lambda)\left[(-\delta_A - \lambda)(-\gamma - \lambda) - \pi(1-\tau)\beta_\epsilon \alpha_S/\delta_S\right] + \tau \beta_\epsilon \pi \xi \alpha_S/\delta_S\right\}. \tag{S9}$$

We immediately identify the eigenvalue $\lambda_1 \approx -\delta_S$. Then, noting that the trailing term $\tau \beta_\epsilon \pi \xi \alpha_S/\delta_S$ is many orders of magnitude smaller than the other quantities (see parameter values in Table 1 of main body), we drop this term and identify a second eigenvalue $\lambda_2 \approx \theta_L$. We remind the reader that $\theta_L = \alpha_L - \delta_L - \xi$ is a negative number and defined as the net clearance rate of the latent reservoir per Ref.[14] Thus, we have identified two negative eigenvalues, meaning that perturbations to the viral free equilibrium return to equilibrium along those respective eigendirections. Solving for the remaining eigenvalues using the expression within the square brackets leads to

$$\lambda_{3/4} = \frac{(\delta_A + \gamma)}{2}\left\{-1 \pm \sqrt{1 - 4\frac{\delta_A \gamma}{(\delta_A + \gamma)^2}\left[1 - \frac{\pi(1-\tau)\beta_\epsilon \alpha_S}{\delta_S \delta_A \gamma}\right]}\right\}. \tag{S10}$$

We define the quantity

$$R_0^{ART} = \frac{\pi(1-\tau)\beta_\epsilon \alpha_S}{\delta_S \delta_A \gamma}, \tag{S11}$$

calling it the basic reproductive number that depends on the ART drug efficacy $\epsilon$. By following through calculations (noting bounds on the factor $4\frac{\delta_A \gamma}{(\delta_A+\gamma)^2} \in [0,1]$) it can be shown that if $R_0^{ART} < 1$ both $\lambda_{3/4} < 0$ such that the viral free equilibrium is stable. Letting $R_0^{ART} = 1$ makes $\lambda_3 = 0$ and $\lambda_4 < 0$, while $R_0^{ART} > 1$ results in $\lambda_3 \geq 0$ while $\lambda_4 < 0$. The basic reproductive number controls the stability of the viral free equilibrium, only when $R_0^{ART} > 1$ will an infection take off. Note that the same result can be verified using the next-generation matrix method[2]

To calculate the "critical drug efficacy" $\epsilon_c$, we solve for the drug efficacy that makes $R_0^{ART} \leq 1$. This yields

$$\epsilon_c \geq 1 - \frac{\delta_S \delta_A \gamma}{\pi \alpha_S (1-\tau)\beta}. \tag{S12}$$

or using the typical definition of the basic HIV dynamics model $R_0 = \frac{\pi \alpha_S \beta}{\delta_S \delta_A \gamma}$, we see that

$$\epsilon_c \geq 1 - \frac{1}{(1-\tau)R_0}. \tag{S13}$$

For our parameter values, $R_0 \sim 8$ as expected for HIV, guaranteeing instability of the viral free equilibrium for any introduction of virus.

Observations from patient data show that primary infections in humans lead to a durable viral setpoint. The Jacobian of our model evaluated at the endemic equilibrium can be written by inserting the equations Eq. S2 into Eq. S7. By calculating the eigenvalues of this Jacobian, we assess the stability of the setpoint equilibrium. Numerical computation of the eigenvalues of this matrix (using `eig()` in Matlab) for varying values of the drug efficacy are presented in Fig. S1. Here we find that above the critical drug efficacy $\epsilon_c$ (for our parameters $\epsilon_c \sim 85\%$), the real part of the third eigenvalue becomes positive. Therefore, while the setpoint equilibrium is stable for low drug levels, $\epsilon < \epsilon_c$, above the critical efficacy the setpoint is no longer stable. At precisely this drug level the viral free equilibrium becomes stable again, which can be calculated numerically, or seen from the analytical derivation above.

Furthermore, calculating the eigenvalues of the viral free equilibrium shows that above the critical efficacy, the viral free equilibrium becomes stable (all eigenvalues having negative real parts). For our parameter range, the eigenvalues (-0.2, -0.0006, -0.3, -24) approximate the death or clearance rates of susceptible cells, latent cells, active cells, and virus respectively. This result agrees with our analytical approximation of the first 2 eigenvalues above. Only the eigenvalue related to the active cell rate is noticeably increased relative to its natural rate, presumably due to coupling between two eigendirections. Most importantly, the disparate timescales allow adiabatic decoupling of the processes.[3] That is, because the solution for the return to equilibrium corresponds to the solution of

$$\frac{d}{dt}\Delta\mathbf{x} \approx \mathbf{J}_{\text{vfe}}\Delta\mathbf{x}, \tag{S14}$$

or using our eigenvalue decomposition, the complete solution to the dynamics of a perturbation from the viral free equilibrium can be expressed as

$$\Delta\mathbf{x} = \sum_j c_j e^{\lambda_j t}\mathbf{v}_j \tag{S15}$$

where $\mathbf{v}_j$ are the 4 eigendirections, not explicitly written here. The timescale for decay of each eigendirection is proportional to $1/\lambda_j$. For example, the fastest eigendirection $\lambda_4$ becomes negligible in the timescale of a day, corresponding to viral clearance. Two of the other eigenvalues have timescales of around 1 week, corresponding to clearance of active cells. However, the time required to completely return to viral free equilibrium ($t^*$) depends on the slowest rate or the smallest magnitude eigenvalue as $t^* \approx 1/\min|\lambda_j|$.[1] In our case, the smallest eigenvalue is approximately $\theta_L$ so that complete return to viral free equilibrium ultimately depends only on the latent cell clearance rate. This justifies our approximation that virus is negligible soon after ART initiation and that the model for long term ART can be simplified to

$$\dot{L} \approx \theta_L L, \tag{S16}$$

which has the solution

$$L = L_0 \exp(\theta_L t). \tag{S17}$$

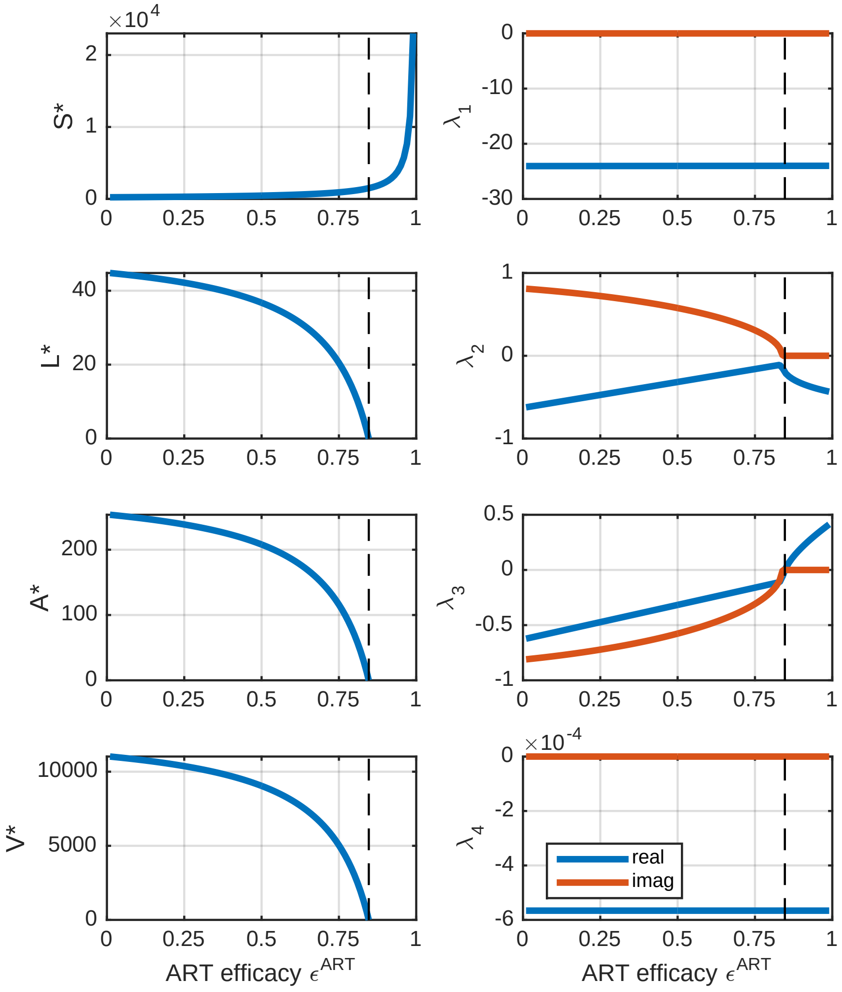


Figure S1: Setpoint stability depends on the efficacy of antiretroviral therapy (ART). Plots of the equilibrium concentrations and the eigenvalues of **J** (Eq. S7) evaluated at those viral setpoint equilibrium (Eq. S3) values at varying ART efficacy $\epsilon^{ART}$. The critical therapy thresholds are illustrated by the vertical dashed line (with our parameters and Eq. S12 we can calculate $\epsilon_c \sim 85\%$). At this value the real part of the third eigenvalue becomes positive making the viral equilibrium unstable.

In summary, our model system rests at the viral free equilibrium prior to infection. Any virus results in HIV infection which progresses and settles at a stable viral setpoint equilibrium. However, ART disrupts this equilibrium, making it unstable and driving the system to return to viral free equilibrium. This movement toward viral-free equilibrium is slow and is limited by the rate of latent cell decay. But, this limiting decay is much slower than the other decays, making it possible to ignore those dynamics, and focus solely on the exponential clearance of the latent cells.

### 1.3 Deriving the basic reproductive number $R_0$ with the next-generation matrix method

Following Diekmann, Heesterbeek, and Roberts,[2] we calculate the basic reproduction number of our complete model we begin by breaking apart our model into 2 matrices in the 'next-generation' (NG) fashion. We assume that the

model is at the uninfected steady state so that $\dot{S} = 0$ and define $S = S_0 = \alpha_S/\delta_S$. Then, in matrix notation, calling $\mathbf{x} = (L, A, V)^T$ we write

$$\frac{d\mathbf{x}}{dt} = (\mathcal{F} - \mathcal{V})\mathbf{x} \tag{S18}$$

where $\mathcal{F}$ is the matrix that describes new infections in each compartment

$$\mathcal{F} = \begin{bmatrix} 0 & 0 & \tau\beta S_0 \\ 0 & 0 & (1-\tau)\beta S_0 \\ 0 & 0 & 0 \end{bmatrix}, \tag{S19}$$

and $\mathcal{V}$ is the matrix that describes the rates for leaving each compartment and for moving between them

$$\mathcal{V} = \begin{bmatrix} -\theta_L & 0 & 0 \\ -\xi & \delta_A & 0 \\ 0 & -\pi & \gamma \end{bmatrix}. \tag{S20}$$

Then we want write the relationship

$$\frac{d\mathbf{x}}{dt} = (\mathcal{F}\mathcal{V}^{-1} - \mathbb{1})\mathcal{V}\mathbf{x} \tag{S21}$$

so that the largest eigenvalue of $\mathcal{M}_{\mathrm{NG}} = \mathcal{F}\mathcal{V}^{-1}$ will be our reproductive number, and we can see that at least 1 eigenvalue must be greater than 1 for an infection to take off. We have

$$\mathcal{V}^{-1} = \begin{bmatrix} -\frac{1}{\theta_L} & 0 & 0 \\ -\frac{\xi}{\delta_A\theta_L} & \frac{1}{\delta_A} & 0 \\ \frac{\pi\xi}{\delta_A\theta_L\gamma} & -\frac{\pi}{\delta_A\gamma} & \frac{1}{\gamma} \end{bmatrix}. \tag{S22}$$

and thus

$$\mathcal{F}\mathcal{V}^{-1} = \begin{bmatrix} -\frac{\pi\xi}{\delta_A\theta_L\gamma}\beta S_0\tau & \frac{\pi}{\delta_A\gamma}\beta S_0\tau & \frac{1}{\gamma}\beta S_0\tau \\ -\frac{\pi\xi}{\delta_A\theta_L\gamma}\beta S_0(1-\tau) & \frac{\pi}{\delta_A\gamma}\beta S_0(1-\tau) & \frac{1}{\gamma}\beta S_0(1-\tau) \\ 0 & 0 & 0 \end{bmatrix}. \tag{S23}$$

The next generation matrix admits two eigenvalues of zero and one that is positive. The positive eigenvalue is the largest eigenvalue of $\mathcal{M}_{NG}$ and therefore we have the basic reproductive number of our model:

$$\mathcal{R}_0 = \frac{\beta S_0\pi}{\gamma\delta_A}[1 - \tau(1 + \xi/\theta_L)]. \tag{S24}$$

Comparing this value to the approximate derivation above (Eq. S11), we see that because $\xi/\theta_L < 1$, the approximation is fairly accurate. Additionally, we could have carried through the calculation using infectivity as altered by ART efficacy without loss of generality.

### 1.4 Allowing direct active to latent transitions has a neglible impact on reservoir size.

The contribution of actively infected cells to latency can be ignored such that we can consider the latent cell equation alone. This follows from a heuristic argument made by Conway and Perelson.[4] On ART, virus can be created by latent cell activation, which occurs rarely. However, the infectivity of HIV is reduced by $\epsilon_c$ on ART such that most susceptible cells are protected from infection. Further, the likelihood of any new infected cell becoming latent is quite small ($\tau$). Thus, the product of the rare events–activation, new infection, and latency–is effectively zero.

Still, in some modeling works a transition from active to latent cells has been included.[5,6] If we add a transition term to the model as a rate $\phi$, the equations for the latent and active pool (decoupled from the virus and susceptible as before) are

$$\begin{aligned} \dot{L} &= \alpha_L L - \delta_L L - \xi L + \phi A \\ \dot{A} &= \xi L - \delta_A A - \phi A. \end{aligned} \tag{S25}$$

We verified numerically that only for unnaturally large values of $\phi > \alpha_L$ (or likewise $\phi > \delta_L$) does including this transition impact the reservoir size. In that case, there is a transient increase in the initial number of latent cells before the typical latent clearance dynamics take hold. This result is a consequence of the rapid death rate of active cells relative to the other rates.

### 1.5 Impact of duration and rates on clearance time

In the main article, we proposed that optimal latent clearance is achieved by increasing the duration of therapy rather than increasing the potency of the therapy equivalently (Fig. 2). If we examine the remaining fraction of latent cells over time $L/L_0 = \exp\left[(\alpha_L - \delta_L - \xi)t\right]$, we can study the effect of multiplying any rate by a factor $r$, denoted $L^{(r)}$ or multiplying the duration of time by factor $d$, denoted $L^{(d)}$. The ratio of the percent remaining after doing either of these multiplications tells us which procedure is more valuable. For example, if we multiply the activation rate only,

$$\frac{L^{(r)}}{L^{(d)}} = \exp\left[t(\alpha_L - \delta_L - r\xi - d(\alpha_L - \delta_L - \xi))\right].$$

Then for $L^{(r)}$ to be smaller than $L^{(d)}$, we must have

$$r > \frac{(\alpha_L - \delta_L)(1-d) + \xi d}{\xi}.$$

Note if we set $d = r$, we find that $d < 1$ because $(\alpha_L - \delta_L)$ is a negative number. Therefore, the decrease in the latent pool due to multiplying the duration of therapy will always be larger than an equivalent multiplication of the rate (unless the $r < 1$, which is a nonsensical proposition equivalent to making therapy less effective).

## 2 Model parameters

### 2.1 Latent cell parameters: $\theta_L$, $\alpha_L$, $\delta_L$, $\xi$

Our most critical parameter values are those that describe the proliferation, death, and activation rates of latently infected cells–the sum of which is their net clearance rate $\theta_L$. Measuring these parameters *in vivo* in humans presents experimental challenges, and results depend on the models chosen to interpret the experimental data.[7] We acknowledge that there is uncertainty in these parameter estimates and thus provide an uncertainty and sensitivity analysis to demonstrate theoretical ranges for plausible outcomes (Fig. 4). Note that despite the variability of proliferation estimates, for even the slowest proliferation estimates we have encountered in the literature, proliferation rates are still two orders of magnitude larger than the estimated activation rate.

We use the proliferation rate $\alpha_L$ from Macallan *et al.* who measured *in vivo* turnover with deuterated glucose labeling in memory T cells in healthy adults and found that on average, 1.5% of CD45R0$^+$CCR7$^+$ T cells (central memory) proliferate daily, whereas 4.7% of CD45R0$^+$CCR7$^-$ T cells (effector memory) and 0.2% of CD45R0$^-$CCR7$^+$ T cells (naïve) proliferate per day, corresponding with exponential growth rates of $\alpha_i$ = 0.015, 0.047, and 0.002, respectively.[8] We use Macallan *et al.*'s estimates from "healthy" adults (*i.e.* HIV-negative) because this study includes proliferation estimates for the CD4$^+$T cell subsets of interest.

In fact, several experimental works have demonstrated similar rates of memory CD4$^+$T cell proliferation among HIV-negative and HIV-infected on long-term ART.[9,10] Whereas these studies examine the entire pool of CD4 T cells, rather than the smaller pool of latently infected cells, there is no empirical evidence to suggest that proliferation rates of latently infected cells would be *lower* than their uninfected counterparts. Indeed, Chomont *et al.* demonstrated that systemic proliferation events are tightly linked to the size and composition of the latent reservoir.[11] These studies show that CD4$^+$T cell proliferation rates are similar among HIV-uninfected and HIV-infected persons on long-term ART. However, net proliferation or proliferation of naïve versus memory cell rates are measured rather than the effector and central memory subsets that are now recognized as being important reservoir subsets, and thus we adopted the Macallan *et al.* rates.

Note that in our model, as in all T cell proliferation references we cite, proliferation rates reflect a combined impact of clonal expansion and homeostatic proliferation, as these are not yet distinguishable experimentally.

To estimate the activation rate of latent cells, Hill *et al.* used values found in Luo *et al.* from several structured treatment interruptions and found that on average, 57 CD4$^+$T cells per day transition from latency to the activated state.[12,13] Assuming that the reservoir contains 1 million cells on average, the rate of activation of a single cell per day $\xi = 5.7 \times 10^{-5}$.[13] Note that the activation rate $\xi$ is several orders of magnitude smaller than the proliferation rate $\alpha_L$.

The net clearance rate of the latent reservoir is estimated from Siliciano *et al.*'s quantitative viral outgrowth assay as $\theta_L$ = -5.2 $\times 10^{-4}$ per day.[14] This result was corroborated by Crooks *et al.*[15]

## 2.2 Susceptible and active cell parameters: $\alpha_S$, $\delta_S$, $\alpha_A$, $\delta_A$

In the complete model, the production of CD4$^+$T cells from the bone marrow and thymus is described by $\alpha_S$; and $\delta_S$ is the rate of susceptible T cell death. In several early HIV modeling papers, a value of 10 per $\mu$L-day was estimated for the production rate[16,17] with $\delta_S$ estimated at 0.02. Luo *et al.* used Bayesian statistical modeling to estimate HIV model parameters using data from 10 patients who underwent a series of 3-5 ART treatment interruptions with viral loads taken three times weekly following interruptions and then weekly following initiation of treatment.[12] They estimated $\alpha_S = 295$, $\delta_S = 0.18$, and $\delta_A = 1$. Huang *et al.* also use Bayesian methods (Markov Chain Monte Carlo) and fit their model to data from Ref.,[18] an AIDS clinical trial comparing dosing regimens for indinavir and ritonavir.[19] Huang *et al.*'s model also incorporated adherence, drug concentrations, and drug susceptibilities. They find $\alpha_S = 98.1$, $\delta_S = 0.08$, and $\delta_A = 0.37$. Note in Table S1 that the ratio of $\alpha_S$ to $\delta_S$ varies between 500–1639 with the more recent experiments in relative agreement near 1500. Thus, we chose $\alpha_S = 300$, $\delta_S = 0.2$ to reflect this ratio.

The death rate of productively infected cells $\delta_A$ was initially thought to be 0.24.[16] Later, Perelson *et al.* find $\delta_A = 0.5$ based on frequent sampling of 5 patients after giving ritonavir monotherapy.[20] Using a fixed viral clearance rate $\gamma = 23$ from,[21] Markowitz *et al.* determined $\delta_A = 1$ based on potent antiretroviral therapy with lopinavir-ritonavir, tenofovir, lamivudine, and efavirenz in 5 chronically-infected patients.[22] We chose $\delta_A = 1.0$ to reflect more recent experimental findings. We assign $\alpha_A = 0$ because the relative rate of proliferation of actively infected cells likely occurs at negligible rates compared to the death rate of these cells.

| Parameter | Perelson[16] | Huang[19] | Luo[12] | Markowitz[22] | Units |
|---|---|---|---|---|---|
| $\alpha_S$ | 10 | 98.1 | 295 | - | per $\mu$L-day |
| $\delta_S$ | 0.02 | 0.08 | 0.18 | - | per day |
| $\alpha_S/\delta_S$ | 500 | 1226 | 1639 | - | per day |
| $\delta_A$ | 0.24 | 0.37 | 1.0 | 1.0 | per day |

Table S1: Proliferation and death parameters for susceptible and actively infected cells.

## 2.3 Estimating the infectivity $\beta$

Perelson *et al.* calculate the infectivity (using physical diffusion) of a virus finding $\beta = 2.4 \times 10^{-5}\mu$L per day.[16] This value is ubiquitous but Luo *et al.* and Huang *et al.* also estimate parameter values for $\beta$ as $3.9\times10^{-3}$ and $1.7\times10^{-5}$, respectively.[12,19] Given that the range of these values spans two orders of magnitude, we chose a value between these extremes: $10^{-4}$.

| Parameter | Perelson[16] | Huang[19] | Luo[12] | Units |
|---|---|---|---|---|
| $\beta$ | $2.4\times10^{-5}$ | $1.7\times10^{-5}$ | $3.9\times10^{-3}$ | $\mu$L per day |

Table S2: Infectivity.

## 2.4 Viral parameters: $\pi$, $\gamma$

The viral 'burst rate' $\pi$ is the amount of virus a single actively infected cell emits in a day (roughly its lifetime). Rong and Perelson note that $\pi$ is problematic, as its experimental value is under question and affects the values of many of the other parameters.[5] Haase *et al.* used radioactively-labeled RNA probes and quantitative image analysis to determine the number of viral particles per mononuclear cell in biopsies from fixed lymph tissue finding a mean value of 74.[23] Using quantitative, competitive, real-time PCR to measure the mean viral RNA copy number per infected cell from fresh-frozen cervical lymph nodes from 9 HIV patients with varied viral loads, Hockett *et al.* found $\pi = 10^{3.6} = 4 \times 10^{3}$.[24] Both of these estimates do not necessarily reflect the number of copies produced in a day per cell or in a cell's lifetime, rather the amount that was being produced at the instant the experiments were performed. We adopt $\pi = 10^{3}$. For the viral clearance rate $\gamma$, we use Ramratnam *et al.*'s estimate $\gamma = 23$, obtained from viral load measurements taken over 5 days before, during, and after apheresis in 4 patients assuming a constant rate of viral production.[21]

| Parameter | Perelson[16] | Huang[19] | Luo[12] | Units |
|---|---|---|---|---|
| $\pi$ | 1200 | 976 | $5.9\times10^{3}$ | copies/cell-day |
| $\gamma$ | 2.4 | 3.06 | 18.8 | per day |
| $\pi/\gamma$ | 500 | 319 | 314 | copies/cell |

Table S3: Viral burst and clearance rates. Note:[12] used the data from[21] but used the geometric mean rather than the arithmetic mean, *i.e.* 18.8 rather than 23.

## 2.5 The latency fraction: $\tau$

The 'latent cell fraction' $\tau$ is the rate at which newly infected cells join the latent cell pool, whereas $1 - \tau$ is the rate at which they join the actively infected pool. The few estimates of this parameter fall throughout a wide range. Despite this, the choice of $\tau$ within the given range does not affect our cure estimates or our estimate of the critical epsilon ($\epsilon_c$). Because Conway and Perelson's estimates are based on modern measurements of the reservoir, we chose $\tau = 10^{-4}$, the upper bound on their estimates.[4]

| Parameter | Callaway[17] | Jones[25] | Conway[4] | Units |
|---|---|---|---|---|
| $\tau$ | $10^{-6}$ | $10^{-3}$ | $10^{-4}$ | unitless |

Table S4: Fraction of infected cells that enter a latent state.

## 2.6 Parameter estimates, ranges, and confidence intervals

See Table S5 for the complete table of parameters that we incorporated in the uncertainty analysis.

| Param. | Value | (CI)/[Literature Range] | Dimensions | Source |
|---|---|---|---|---|
| $\alpha_S$ | 300 | [10, 760] | cells per $\mu$L-day | [12,16,19] |
| $\delta_S$ | 0.2 | [0.02, 0.45] | per day | [12,16,19] |
| $\alpha_{cm}$ | 0.015 | (0.01, 0.02) | per day | [8] |
| $\delta_{cm}$ | 0.0155* | | per day | calculated* |
| $\alpha_{em}$ | 0.047 | (0.038, 0.057) | per day | [8] |
| $\delta_{em}$ | 0.0475* | | per day | calculated* |
| $\alpha_n$ | 0.002 | (0.0015, 0.0023) | per day | [8] |
| $\delta_n$ | 0.0025* | | per day | calculated* |
| $\delta_A$ | 1.0 | (0.8, 1.2) | per day | [12,22] |
| $\xi$ | $5.7 \times 10^{-5}$ | $(5.4,\ 6.0) \times 10^{-5}$ | per day | [13] |
| $\theta_L$ | $-5.2 \times 10^{-4}$ | $-(2,\ 8.4) \times 10^{-4}$ | per day | [14] |
| $\beta$ | $1 \times 10^{-4}$ | $[0.016,\ 6] \times 10^{-3}$ | $\mu$L/copy-day | [12,16,19] |
| $\tau$ | $10^{-4}$ | $[10^{-6},\ 10^{-3}]$ | unitless | [4,17,25] |
| $\pi$ | $10^3$ | [20, 5340] | copies/cell-day | [12,16,19] |
| $\gamma$ | 23 | [9, 36] | per day | [21] |
| $L_0$ | $10^6$ | $[10^4,\ 10^8]$ | cells | [14] |
| $L_{cm}(0)$ | $0.6L_0$ | $[0.2,\ 0.8]L_0$ | cells | [11,26] |
| $L_{em}(0)$ | $0.4L_0$ | $[0.2,\ 0.8]L_0$ | cells | [11,26] |
| $L_n(0)$ | $0.02L_0$ | $[0,\ 0.1]L_0$ | cells | [11,26] |

Table S5: A summary of all parameters used in our simulations. $^*\delta_L$ is back-calculated from known $\alpha_L$, $\theta_L$, and $\xi$. 95% confidence intervals are given in parentheses () where applicable from experimental data. Otherwise, the range is taken from our literature search or from the ranges given in the cited works not assumed to be normally distributed; these values are given in square brackets [].

# 3 Mycophenolate mofetil (MMF)

## 3.1 Determining the Hill coefficient and IC50s

In dose-response relationships, the concentration of drug that inhibits the response by 50% is called the IC50. The slope at the steepest point along the dose-response curve is called the Hill slope (or coefficient).[27]

The Hill coefficient and the IC50s for MMF were calcuated using the `drc` package in the `R` statistical computing language. Specifically, the `drm` fitting command was used to fit the experimental *in vitro* proliferation data to the 'LL.4' (four parameter log-logistic function) function:

$$f(x) = c + \frac{d - c}{1 + (\frac{x}{IC50})^m} \tag{S26}$$

in which $f(x)$ represents the fraction of proliferating cells at mycophenolic acid (MPA) concentration $x$. The upper limit of cellular proliferation in the experiment is $c$ (no drug) and $d$ is the lower limit (maximal drug effect).[28]

The active metabolite of MMF is mycophenolic acid, which was used in the titration experiments.

## 3.2 Previous studies in HIV-infected patients treated with ART and MMF

MMF has been given to HIV-infected patients in various settings, either experimentally as an antiviral drug or as part of standard regimens after kidney transplantation. Below we review the studies in which MMF was given to HIV-infected patients and either viral load reduction, reservoir reduction as measured by viral co-culture (QVOA), or time to viral rebound after treatment cessation were measured. Two trials assessed reservoir reduction using viral co-culture. Chapuis *et al.* revealed a reduction of the viral reservoir in the ART and MMF combination group but no reduction in the group on ART alone.[10] Sankatsing *et al.* also studied MMF and ART combination treatment and found a

mean daily decay rate of latently infected cells (using viral co-culture) of 0.017 infected cells/$10^6$ cells in patients on MMF and 0.004 infected cells/$10^6$ cells in patients on ART alone. Despite the fact that this trend was not statistically significant(only eight patients in the MMF + ART group and nine in the ART-only group), the reservoir decay rate in the MMF-treated patients was almost five times as high as in the ART-only patients.

Both García *et al.* and Millán *et al.* demonstrated a delay in viral rebound after cessation of ART in patients who received MMF in addition to ART of 2-3 weeks.[29,30]

Margolis *et al.* gave MMF to five HIV-infected patients failing antiretroviral therapy with an average viral load of $10^{4.78}$ copies/mL. Four of the five patients experienced a greater than 0.5 log decrease in viral load compared to entry whereas three of five sustained the 0.5 log decrease after one year on ART combined with MMF.

Jurriaans *et al.* published a case of an HIV-infected patient who received a five drug ART regimen plus MMF and sero-reverted.[31]

# References


[1] Strogatz, S. H. *Nonlinear dynamics and chaos: with applications to physics, biology, chemistry, and engineering* (Westview press, 2014).

[2] Diekmann, O., Heesterbeek, J. & Roberts, M. The construction of next-generation matrices for compartmental epidemic models. *J Roc Soc Interface* **7**, 873–885 (2010).

[3] Pathria, R. K. Statistical mechanics. (1972).

[4] Conway, J. & Perelson, A. Residual Viremia in Treated HIV+ Individuals. *PLoS Comput Biol* **12**, e1004677; 10.1371/journal.pcbi.1004677 (2016).

[5] Rong, L. & Perelson, A. Modeling latently infected cell activation: Viral and latent reservoir persistence, and viral blips in HIV-infected patients on potent therapy. *PLoS Comput Biol* **5**, e1000533; 10.1371/journal.pcbi.1000533 (2009).

[6] Ribeiro, R., Mohri, H., Ho, D. & Perelson, A. *In vivo* dynamics of T cell activation, proliferation, and death in HIV-1 infection: why are CD4+ but not CD8+ T cells depleted? *Proc Natl Acad Sci USA* **99**, 15572–15577 (2002).

[7] De Boer, R. & Perelson, A. Quantifying T lymphocyte turnover. *J Theor Biol* **327**, 45–87 (2013).

[8] Macallan, D. C. *et al.* Rapid turnover of effector-memory CD4+ T cells in healthy humans. *J Exp Med* **200**, 255–260 (2004).

[9] Mohri, H. *et al.* Increased turnover of T lymphocytes in HIV-1 infection and its reduction by antiretroviral therapy. *J Exp Med* **194**, 1277–1287 (2001).

[10] Chapuis, A. G. *et al.* Effects of mycophenolic acid on human immunodeficiency virus infection *in vitro* and *in vivo*. *Nat Med* **6**, 762–768 (2000).

[11] Chomont, N. *et al.* HIV reservoir size and persistence are driven by T cell survival and homeostatic proliferation. *Nat Med* **15**, 893–900 (2009).

[12] Luo, R., Piovoso, M., Martinez-Picado, J. & Zurakowski, R. HIV model parameter estimates from interruption trial data including drug efficacy and reservoir dynamics. *PLOS ONE* **7**, e40198; 10.1371/journal.pone.0040198 (2012).

[13] Hill, A., Rosenbloom, D., Fu, F., Nowak, M. & Siliciano, R. Predicting the outcomes of treatment to eradicate the latent reservoir for HIV-1. *Proc Natl Acad Sci USA* **111**, 15597; 10.1073/pnas.1406663111 (2014).

[14] Siliciano, J. D. *et al.* Long-term follow-up studies confirm the stability of the latent reservoir for HIV-1 in resting CD4+ T cells. *Nat Med* **9**, 727–728 (2003).

[15] Crooks, A. M. *et al.* Precise quantitation of the latent HIV-1 reservoir: implications for eradication strategies. *J Infect Dis* **212**, 1361–1365 (2015).

[16] Perelson, A. S., Kirschner, D. E. & De Boer, R. Dynamics of HIV infection of CD4+ T cells. *Math Biosci* **114**, 81–125 (1993).

[17] Callaway, D. & Perelson, A. HIV-1 infection and low steady state viral loads. *Bull Math Biol* **64**, 29–64 (2002).

[18] Acosta, E. P. *et al.* Comparison of two indinavir/ritonavir regimens in the treatment of HIV-infected individuals. *J Acquir Immune Defic Syndr* **37**, 1358–1366 (2004).

[19] Huang, Y., Liu, D. & Wu, H. Hierarchical Bayesian methods for estimation of parameters in a longitudinal HIV dynamic system. *Biometrics* **62**, 413–423 (2006).

[20] Perelson, A., Neumann, A., Markowitz, M., Leonard, J. & Ho, D. HIV-1 dynamics in vivo: virion clearance rate, infected cell life-span, and viral generation time. *Science* **271**, 1582–1586 (1996).

[21] Ramratnam, B. *et al.* Rapid production and clearance of HIV-1 and hepatitis C virus assessed by large volume plasma apheresis. *Lancet* **354**, 1782–1785 (1999).

[22] Markowitz, M. *et al.* A novel antiviral intervention results in more accurate assessment of HIV-1 replication dynamics and T-cell decay *in vivo*. *J Virol* **77**, 5037–5038 (2003).

[23] Haase, A. T. *et al.* Quantitative image analysis of HIV-1 infection in lymphoid tissue. *Science* **274**, 985–989 (1996).

[24] Hockett, R. D. *et al.* Constant mean viral copy number per infected cell in tissues regardless of high, low, or undetectable plasma HIV RNA. *J Exp Med* **189**, 1545–1554 (1999).

[25] Jones, L. & Perelson, A. Transient viremia, plasma viral load, and reservoir replenishment in HIV-infected patients on antiretroviral therapy. *J Acquir Immune Defic Syndr* **45**, 483–93 (2007).

[26] Buzon, M. J. *et al.* HIV-1 persistence in CD4+ T cells with stem cell-like properties. *Nat Med* **20**, 139–142 (2014).

[27] Gadagkar, S. & Call, G. Computational tools for fitting the hill equation to dose-response curves. *J Pharmacol Toxicol Methods* **71**, 68–76 (2015).

[28] Ritz, C., Baty, F., Streibig, J. C. & Gerhard, D. Dose-response analysis using R. *PLOS ONE* **10**, e0146021; journal.pone.0146021 (2015).

[29] García, F. *et al.* Effect of mycophenolate mofetil on immune response and plasma and lymphatic tissue viral load during and after interruption of highly active antiretroviral therapy for patients with chronic HIV infection: a randomized pilot study. *J Acquir Immune Defic Syndr* **36**, 823–830 (2004).

[30] Millan, O. *et al.* Pharmacokinetics and pharmacodynamics of low dose mycophenolate mofetil in HIV-infected patients treated with abacavir, efavirenz and nelfinavir. *Clin Pharmacokinet* **44**, 525–538 (2005).

[31] Jurriaans, S. *et al.* HIV-1 seroreversion in an HIV-1-seropositive patient treated during acute infection with highly active antiretroviral therapy and mycophenolate mofetil. *AIDS* **18**, 1607–1608 (2004).
